# Accelerated Self-Healing and Property Recovery in Brush Particle Solids Featuring Brush Dispersity

**DOI:** 10.1021/acsmacrolett.5c00036

**Published:** 2025-03-07

**Authors:** Hanshu Wu, Yunping Shi, Ting-Chih Lin, Ayesha Abdullah, Michael R. Bockstaller, Krzysztof Matyjaszewski

**Affiliations:** † Chemistry Department, Carnegie Mellon University, 4400 Fifth Avenue, Pittsburgh, Pennsylvania 15213, United States; ‡ Department of Materials Science and Engineering, Carnegie Mellon University, 5000 Forbes Avenue, Pittsburgh, Pennsylvania 15213, United States

## Abstract

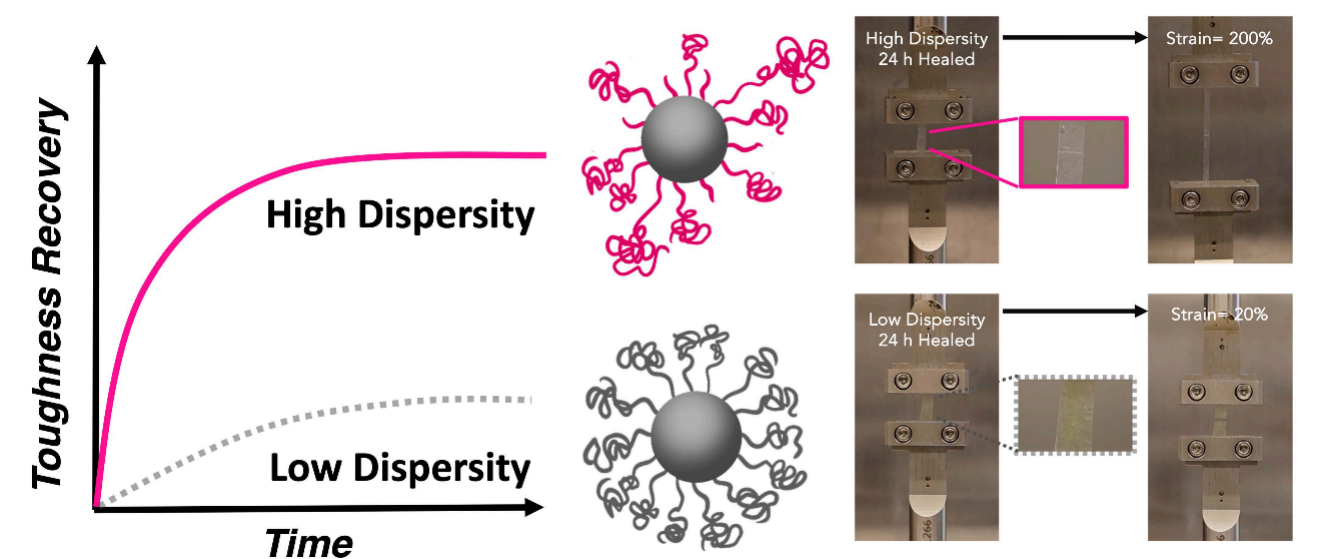

Brush particles, hybrid materials consisting of polymer chains tethered to particle surfaces, offer tunable properties that make them promising candidates for advanced functional materials. This study investigated the role of chain dispersity in the viscoelastic self-healing of poly (methyl acrylate) (PMA)-based brush particle solids. Increasing the molecular weight dispersity of grafted chains significantly enhanced both strain-to-fracture and toughness of brush particle solids, while the elastic modulus and glass transition temperature were independent of chain dispersity. Cut-and-adhere testing revealed a significant acceleration of the rate of toughness recovery in high-dispersity systems as compared to low-dispersity analogs for which toughness recovery markedly lagged the recovery of Young’s modulus. The results suggest that structure and property recovery in brush particle solids are sensitive to the dynamical heterogeneity of brush canopies and highlight the role of molecular weight dispersity as a design parameter to enable hybrid materials with advanced self-healing ability.

The need to increase longevity and sustainability has fueled interest in polymer-based materials that are capable of “self-healing” after incurring structural damage.^1−8^ In general, the recovery of mechanical properties after incurring damage is contingent on the re-establishment of the polymer’s entanglement network structure across the damaged region.^9−12^ In homogeneous thermoplastics, this is commonly accomplished in the rubbery regime when chain dynamics is fast enough to facilitate interdiffusion of chains on practical time scales, thus enabling the welding of interfaces.^3,12−14^ In amorphous polymers, chain dynamics is negatively correlated with the elastic modulus, and this prevents the realization of high-modulus polymers featuring viscoelastic self-healing at ambient temperature (i.e., below the polymer’s glass transition temperature, *T*_g_). Strategies such as the integration of monomer-filled reservoir structures into a polymer host (aka “extrinsic self-healing”) or chemical motifs enabling reversible covalent or noncovalent network structures (aka “intrinsic self-healing”) have been shown to accelerate healing in rigid materials; however, they do not alleviate the need of viscoelastic recovery to achieve full recovery.

We recently demonstrated that a polymer’s ability to “heal” via viscoelastic recovery is retained when grafted from particle surfaces.^15^ Poly(butyl acrylate-*stat*-methyl methacrylate) statistical copolymers (P(BA-*stat*-MMA)) were grafted from silica nanoparticles (size *d* ∼ 15.5 ± 3.7 nm) by using surface-initiated atom transfer radical polymerization (SI-ATRP). Assembly of the copolymer brush particles resulted in brush particle solids featuring higher modulus than the linear polymer analogs (15.6 vs 8.2 MPa) with retained viscoelastic recovery. However, a significant slowdown of the rate of toughness recovery (measured as time needed to reconstitute one-half of the pristine material property) was observed when compared with a linear reference copolymer, whereas the recovery rate of Young’s modulus was unchanged. The slowdown of toughness recovery was attributed to the reduced mobility of surface tethered chains, which constrained the reformation of entanglement network structures. This presents a challenge to applications of self-healing hybrid materials, as it requires higher temperatures (or larger time scales) to initiate healing after damage.

Opportunities to modulate the dynamic properties of polymers arise from deliberate control of the molecular dispersity of grafted chains. For example, for linear amorphous polymers, increasing dispersity resulted in the broadening of the distribution of dynamical processes while local segmental relaxation (which is relevant to the glass transition) remained mostly unchanged.^16^ Since the presence of faster dynamical processes conceivably could result in accelerated recovery, this motivated us to elucidate the effect of molecular dispersity on the viscoelastic recovery rate of brush particle solids. Dispersity has emerged as a key factor influencing the mechanical performance, self-assembly, and processability of these materials.^17−22^ Recent advances in controlled radical polymerization (CRP) have enabled precise control of dispersity.^23−29^ For example, tuning catalyst concentrations in ATRP allows efficient synthesis of high-dispersity polymers.^30,31^ These high-dispersity brush particles exhibited enhanced interparticle entanglements and stronger particle–matrix interactions, resulting in improved mechanical properties.^25,32−34^

To investigate the effect of dispersity on the properties and recovery behavior of brush particle solids, we synthesized analog pairs of low/high dispersity poly (methyl acrylate) (PMA)/silica (SiO_2_) brush particle systems with similar number-average molecular weight (*M*_n_) but selectively varied dispersity (*M*_w_/*M*_n_) using SI-ATRP. Poly (methyl acrylate) was chosen as a model brush system (as opposed to P(BA-*stat*-MMA)) because of its established viscoelastic recovery at room temperature (21 °C), the viability of ATRP to control dispersity in acrylate homopolymer systems, and the absence of convoluting parameters such as monomer sequence distribution and “lock-and-key” interactions that could hinder interpretation of results in copolymer systems.^5,13^ The dispersity of the polymer brushes was tuned by the initial ATRP deactivator concentration ([Cu^II^/L]_0_) in activators regenerated by electron transfer (ARGET)-ATRP process, as described elsewhere.^30,35,36^ Low dispersity samples were synthesized with a higher catalyst concentration of [Cu^II^/L]_0_ = 400 ppm vs monomer, and high dispersity samples were synthesized with a low catalyst concentration of [Cu^II^/L]_0_ = 0.2 ppm. For clarity, brush particles with low-dispersity PMA brushes are denoted as SiO_2_–PMA_*X*_, and those with high-dispersity PMA brushes are denoted as SiO_2_-*dis*-PMA_*X*_, where *X* represents the average degree of polymerization (*N*) rounded to the nearest hundred. Additionally, a linear PMA sample with low dispersity was synthesized via ARGET-ATRP with a high catalyst concentration ([Cu^II^/L] _0_ = 400 ppm) and is referred to as PMA_600_, were 600 is the average *N* rounded to the nearest hundred. Synthetic parameters and relevant details of all of the materials are presented in Table 1.

**Table 1:** Characteristics of SiO_2_-*g*-PMA Brush Particles and Linear PMA

entry^a^	*M*_n_^b^	*M*_w_/*M*_n_^b^	*f*_SiO2_ (%)^c^	σ (nm^–2^)^d^	*T*_g_ (°C)^e^	*T*_g_ range (°C)^e^
SiO_2_-PMA_400_	37260	1.05	17.17	0.45	17	12–22
SiO_2_-PMA_700_	66290	1.06	12.51	0.36	18	11–23
SiO_2_-PMA_900_	82420	1.20	10.76	0.36	18	11–23
SiO_2_-*dis*-PMA_400_	36900	2.19	14.18	0.57	21	16–25
SiO_2_-*dis*-PMA_700_	60620	1.63	13.89	0.36	19	14–23
SiO_2_-*dis*-PMA_900_	81250	2.05	24.64	0.13	16	11–21
PMA_600_	51080	1.08			19	11–24

a Reaction conditions are listed in the Supporting Information.

b Determined by SEC.

c Determined by thermogravimetric analysis (TGA).

d Calculated by eq S1.

e The glass transition temperatures were determined by DSC (Figure S2).

To analyze the molecular weight distribution of PMA chains tethered to silica particles, the samples were etched in hydrofluoric acid solution (CAUTION: HF is considered a highly hazardous chemical and needs to be handled with care) and characterized using size exclusion chromatography (SEC). The molecular weight distributions of the tethered PMA chains are presented in Figure 1a. All samples displayed a monomodal distribution with significant broadening observed in the high dispersity samples. From the SEC traces, the number distribution of the molecular weight was calculated via *n*(*M*_*i*_) ∼ *P*(*M*_*i*_)/*M*_*i*_, where *n*(*M*_*i*_) is the number frequency of the molecular weight, *M*_*i*_ and *P*(*M*_*i*_) are the molecular weight and the volume intensity of *M_i_* (determined using a refractive index detector). As expected for controlled radical polymerization, the number distribution trace (Figure 1b) was positively skewed. A pronounced tailing in the high molecular weight (HMW) range (*M*_n_ > 500000) was observed for high-dispersity samples, a feature that was absent in the low-dispersity counterparts. Similar to previous studies on bimodal brush particle systems,^37^ TEM micrographs (Figure S1) revealed a similar microstructure and scaling of particle-to-particle distance with *M*_w_.

**Figure 1:**
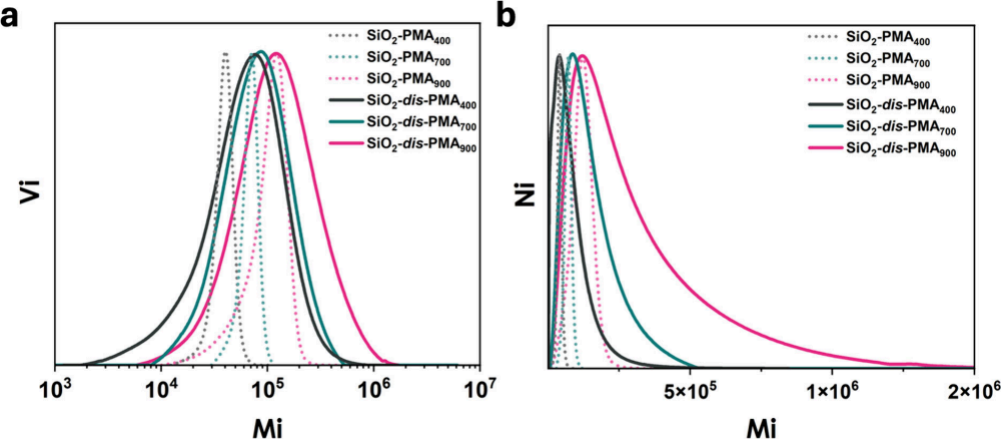
(a) Normalized experimental SEC elution curve measured using refractive index detector in THF after etching of SiO_2_-PMA_*X*_ using HF. (b) Normalized number-weighted distribution of molecular weight.

The impact of brush dispersity on the deformation behavior of SiO_2_-PMA brush particle solids was evaluated by tensile testing (Figure 2a). Young’s modulus (*E*) was calculated from the slope of stress–strain curves in the small-strain regime (ε < 0.01) and was independent of dispersity (Figure 2b). This was attributed to the dominating influence of short-ranged dispersion interactions on Young’s modulus. Since dispersion interactions involve resonance volumes corresponding to only a few repeat units, chain length variation was not expected to impact *E.* In contrast, the toughness (*U*) of films (calculated by integrating stress–strain curves) was significantly larger for disperse brush particle solids (Figures 2c and S3). For instance, SiO_2_-*dis*-PMA_400_ (*U* = 10.44 MPa) and SiO_2_-*dis*-PMA_700_ (*U* = 16.71 MPa) displayed nearly double the toughness of SiO_2_-PMA_400_ (*U* = 4.21 MPa) and SiO_2_-PMA_700_ (*U* = 6.24 MPa). Similarly, SiO_2_-*dis*-PMA_900_ (*U* = 35.92 MPa) featured 1.5 times the toughness of SiO_2_-PMA_900_ (*U* = 24.18 MPa). The trend resembled the increase in toughness reported for bimodal brush particle solids.^37^ We attributed the enhanced *U* of the high-dispersity samples to the more effective entanglement network formation of the high molecular weight PMA fraction that promotes dissipative processes, such as crazing. In low-dispersity samples, the absence of a HMW fraction limits polymer chain entanglement, resulting in chain disentanglement between adjacent brush particles and subsequent material fracture at comparatively low strain.

**Figure 2:**
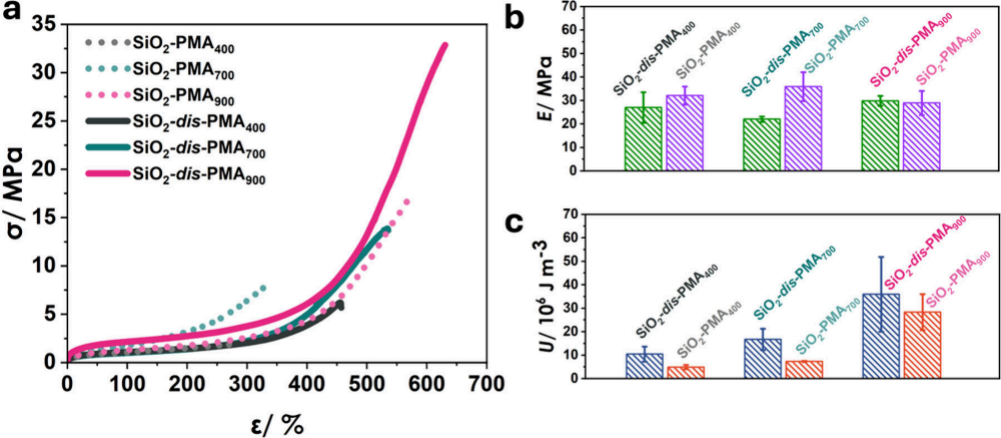
(a) Stress (σ)–strain (ε) curves of low (dotted lines) and high (solid lines) dispersity SiO_2_-*g*-PMA_*X*_ brush particle systems. (b) A comparison chart of Young’s modulus of SiO_2_-*g*-PMA_*X*_ samples. (c) A comparison chart of toughness of SiO_2_-*g*-PMA_*X*_ samples.

To determine the effect of brush dispersity on the viscoelastic properties of materials, we performed dynamic mechanical analysis (DMA) and tensile creep testing. Figure 3a displays the frequency dependence of the loss tangent (tan δ) of brush particle materials and the linear PMA reference system. The peak of tan(δ) was interpreted as the characteristic frequency of the α-relaxation of the brush chains (Figure 3b). The trend in max[tan(δ)] aligned with the near-identical glass transition temperatures of all samples (Table 1, Figure S2) and supported the notion that dispersity had only a weak influence on the local brush relaxation dynamics as well as the glass transition. The observed trend supports previous simulations that elucidated the dynamical properties of disperse polymer melts and concluded that dispersity has a negligible impact on the local chain dynamics (and *T*_g_), but rather increases the dynamic heterogeneity (i.e., the distribution of dynamical processes) in a polymer.^32^

**Figure 3:**
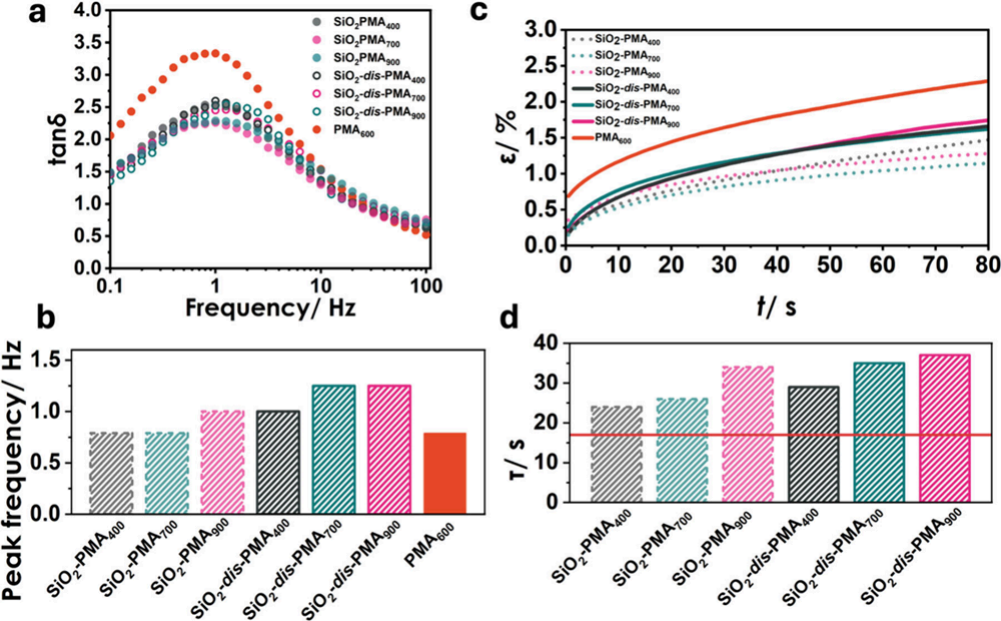
Local and macroscopic dynamical properties of linear PMA and brush particle (SiO_2_-*g*-PMA_*X*_) materials measured at room temperature using dynamic mechanical analysis (a, b) and creep testing (c, d), respectively. (a) Near-identical peak position in loss tangent (tan δ) reveals similar local dynamics of linear and tethered chains. (b) Comparison of characteristic local relaxation times. (c) Time-dependent strain (creep) of a linear, high-dispersity brush particle (solid lines) and a low-dispersity brush particle (dotted lines). Film dimensions for creep testing: width × length × height: 5 mm × 15 mm × 0.15 mm. Corresponding step-function stress curves during creep measurements are shown in Figure S4. (d) Comparison of retardation time derived from creeping results. The red line marks the retardation time of PMA_600_ (τ = 17 s).

As was shown previously, the macroscopic deformation of brush particle solids is strongly influenced by the slow cooperative motion of the brush particle cores.^38,39^ To gain insight into the macroscopic dynamic properties of brush particle solids, tensile creep testing was performed. Bulk films were subjected to a constant uniaxial stress (10 kPa) and the time-dependent strain was measured at 23 °C. Testing conditions were chosen to ensure negligible hysteresis at the applied stress level, and results were independently confirmed for select compositions using shear tests at 100 Pa (Figure S5). As shown in Figure 3c, high-dispersity brush materials featured significantly larger strain amplitudes compared to those of their low-dispersity counterparts. To determine the characteristic time scale of creep, the retardation time (τ) was determined for each material using a standard linear solid analysis.^40^Figure 3d reveals that the retardation time increased with the average molecular weight of grafted chains. This was expected since the mobility of brush particles should decrease with the *N* and entanglement of grafted chains, which place constraints on the macroscopic dynamics. Interestingly, for each brush particle pair, τ was larger for the disperse system as compared to its more uniform analog. This suggests that diffusive displacements and plastic deformation were more prominent in low-dispersity brush particle solids. This could be attributed to the positive skewness of molecular weight distributions, resulting in a small fraction of high-molecular chains that contribute disproportionately to the entanglement network structure and thus further constrain equilibrium formation under stress.

To gain insight into the role of dispersity on the ability of materials to self-heal, cut-and-adhere experiments were performed, following established procedures.^31,38^ Severed bulk films were rejoined and annealed at 100 °C for a specified time before being cooled to room temperature. Test conditions were chosen to allow full recovery of most materials to occur on a practical time scale. Recovery efficacy was quantified by the fractional recovery of elastic modulus (*P*_E_), fracture toughness (*P*_U_), and fracture strain (*P*_ε_) through tensile testing (Figure S6). The half-time of recovery (*t*_1/2_), defined as the time required to recover 50% of the initial value, was used to evaluate the rate of recovery, as summarized in Table S1. Figure 4 displays the evolution of *P*_E_ and *P*_U_ for low (Figure 4a,b) and high (Figures4d,e) dispersity systems.^13,14^

**Figure 4:**
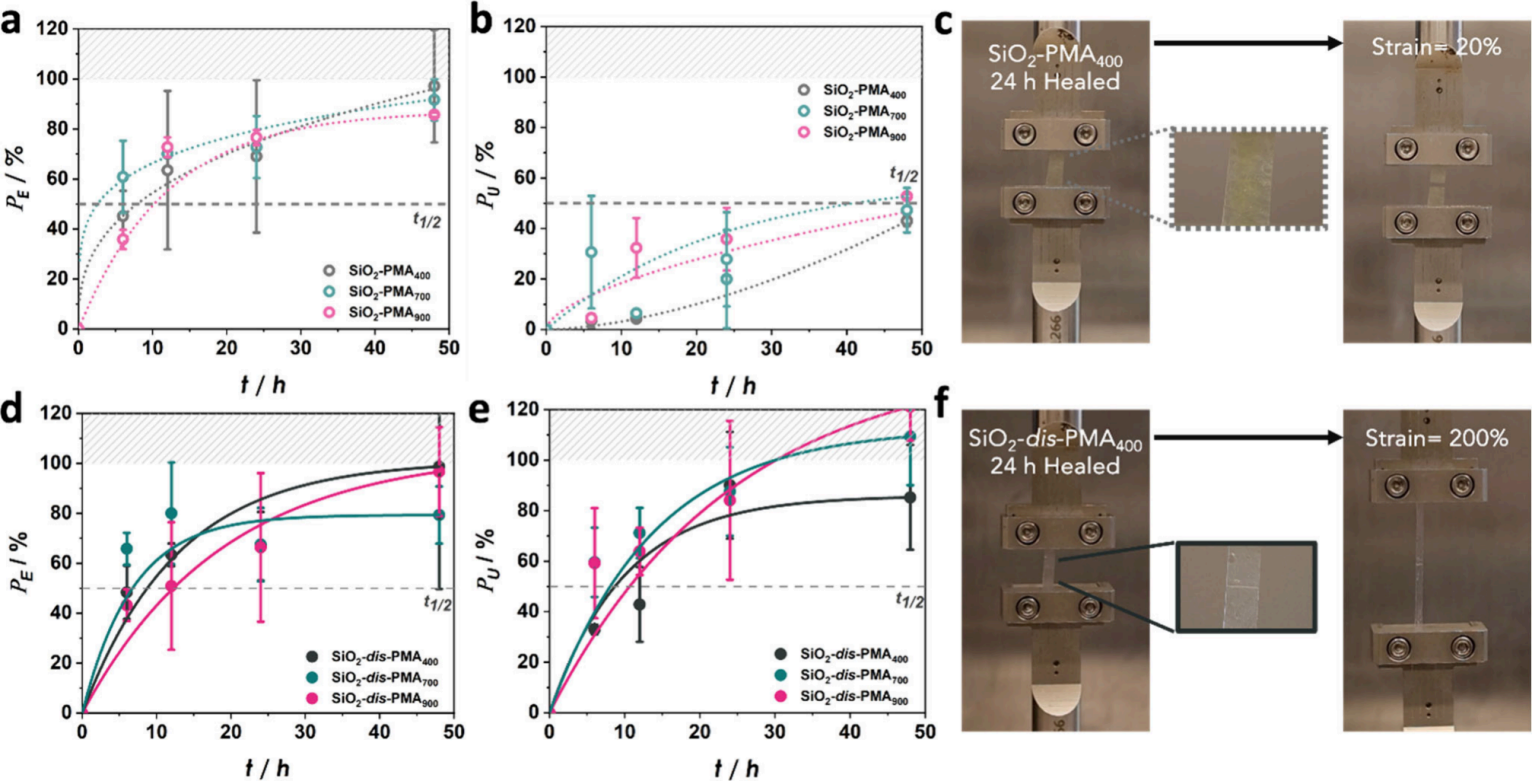
Property recovery of SiO_2_-*g*-PMA_*X*_ samples after rejoining of films and subsequent annealing at 100 °C. Fractional recovery of (a) Young’s modulus (*P*_E_) and (b) toughness (*P*_U_) of low-dispersity samples; (c) Photographs showing a low fracture strain of 20% of SiO_2_-PMA_400_ after rejoining and annealing for 24 h; Fractional recovery of (d) Young’s modulus (*P*_E_) and (e) toughness (*P*_U_) of high-dispersity samples; (f) Photographs showing 200% extensibility of SiO_2_-*dis*-PMA_400_ after rejoining and annealing for 24 h. Values in (a), (b), (d), and (e) are normalized with respect to pristine film properties. Lines are introduced to guide the eye.

Several pertinent trends can be deduced from the data shown in Figure 4. First, both low- and high-dispersity systems featured a comparable half-time for recovery of the elastic (Young’s) modulus of *t*_1/2_ ∼ 7 h, irrespective of the degree of polymerization of tethered chains. The result supported previous conclusions that the elastic modulus is determined by dispersion interactions between surface-grafted chains.^41^ Since dispersion interactions are short-ranged, recovery of Young’s modulus should not require long-range diffusion or chain entanglement. A corollary is that modulus recovery should correlate with the local segmental dynamics of polymer chains, which was indeed supported by the similar glass transition temperatures (Table 1) as well as relaxation times (Figure 3b) of the different brush systems. Second, and in contrast to the recovery trend of Young’s modulus, the rate of toughness recovery featured prominent differences between low- and high-dispersity brush particle materials. For low-dispersity systems, Figure 4b reveals that toughness recovery was significantly delayed with *t*_1/2_ ∼ 50 h for all systems. None of the low-dispersity materials recovered to values greater than 50% within the tested time range. The reduced rate of toughness recovery was consistent with previous reports of self-healing in copolymer-grafted brush particle solids. In analogy to these prior studies, we attributed the prolonged recovery to constrained long-range diffusion processes that are required to re-establish the entanglement network structures that determine the toughness of polymers.^15^ Interestingly, the recovery of toughness in high-dispersity systems occurred at a significantly higher rate and was comparable to the recovery rate of Young’s modulus in the same materials. For instance, in SiO_2_-*dis*-PMA_400_, both *t*^*E*^_1/2_ and *t*^*U*^_1/2_ were about 9 h. No distinctive influence of the degree of polymerization could be discerned, although data in Figure 4e suggest that the toughness recovery rate was somewhat increased for higher molecular systems (SiO_2_-*dis*-PMA_700_ and SiO_2_-*dis*-PMA_900_). The significant acceleration of recovery in high-dispersity brush particle solids could be attributed to several contributing factors that cannot be further differentiated within the present study. Molecular dynamics (MD) simulation of dynamical processes in linear polymer melts established that increasing chain dispersity resulted in a broadening of the distribution of dynamical processes whereas local dynamics remained unchanged.^16^ While no comparable studies have been published for brush particle systems, the effect of dispersity on chain packing in brush systems was evaluated using MD simulation.^42^ A reduction of packing constraints in disperse brushes was reported, which resulted in less efficient packing and a more uniform distribution of chain ends across the brush thickness. It is conceivable that a more relaxed packing environment promotes dynamic heterogeneity in a way similar to what was observed in melts of disperse linear polymers. Thus, our results could indicate that, for brush particle solids with similar local relaxation, toughness recovery is positively correlated with dynamic heterogeneity as well as brush interdigitation which should be more favorable in disperse brush architectures (as deduced from Figure 3d). To further confirm the positive correlation of recovery rate and dispersity, we synthesized and tested a brush particle system with an intermediate dispersity *M*_w_/*M*_n_ = 1.33 and comparable average molecular weight *M*_n_ = 36840 (SiO_2_-*mid-dis*-PMA_400_; sample characteristics shown in Figure S8). The recovery curves (Figure S9) reveal that SiO_2_-*mid-dis*-PMA_400_ featured a similar rate of recovery of Young’s modulus (*t*^*E*^_1/2_ ∼ 7 h), while the rate of toughness recovery (*t*^*U*^_1/2_ ∼ 43 h) was in between those of narrow and high dispersity analogs. It is hoped that these results will motivate future simulation studies to better understand the impact of chain dispersity on the viscoelastic properties and recovery rate in brush particle solids.

In conclusion, the molecular weight dispersity of surface grafted chains has a profound influence on the tensile properties, viscoelastic behavior, and self-healing efficiency of brush particle materials. Toughness, strain-to-fracture, retardation time, and recovery rate were positively correlated with dispersity, whereas local relaxation and the glass transition temperature were not sensitive to changes in dispersity. Building off conclusions from prior research on the effect of dispersity on the viscoelastic properties of polymer blends, our results suggest that, for a given polymer graft composition (i.e., at constant *T*_g_), the rate of self-healing increases with the distribution of dynamic processes, which favors brush interdigitation and entanglement network reformation. The results inform design strategies for the future development of advanced self-healing polymeric materials with increased recovery rates and highlight the importance of synthetic methods to deliberately control the dispersity in polymeric systems.

## Supporting Information Available

Details of synthesis procedures, characterization procedures (DSC, tensile test, DMA, self-healing test, adhesive tests), derivative heat flow curves, strain–stress, and self-healing data (PDF)
