# Supplementary material for: Accelerated Self-Healing and Property Recovery in Brush Particle Solids Featuring Brush Dispersity
Source: ACS Macro Lett. 2025 Mar 7;14(3):371–6. doi: 10.1021/acsmacrolett.5c00036 (PMC11924331; doi:10.1021/acsmacrolett.5c00036)
Supplement: Supplementary file 1 — mz5c00036_si_001.pdf [file mz5c00036_si_001.pdf]

Supporting Information

Accelerated Self-Healing and Property Recovery in Brush Particle Solids Featuring Brush Dispersity

*Hanshu Wu, Yunping Shi, Ting-Chih Lin, Ayesha Abdullah, Michael R. Bockstaller *, Krzysztof Matyjaszewski **

Supporting text

Materials.

Silica particles with ATRP initiating sites (SiO_2-Br) were prepared as reported[1, 2]. Ethyl α-bromoisobutyrate (EBiB, 98%, SigmaAldrich), anisole (99%, Aldrich), tetrahydrofuran (THF, 99%, VWR), methanol (99%, VWR), tris(2-dimethylaminoethyl) amine (Me_6TREN, 99%, Alfa), copper(II) bromide ($CuBr_2$, 99%, Aldrich), tin(II) 2-ethylhexanoate ($Sn(EH)_2$, 95%, Aldrich), N,N-dimethylformamide (DMF, certified, Fisher Chemical). Monomers: methyl acrylate (MA, 99%, Aldrich) was purified by passing through a column filled with basic alumina to remove the inhibitor. Alumina (basic, Super I, 50-200 μm, Sorbtech).

Synthesis of linear polymer and particle brushes.

Initiators (EBiB/SiO_2-Br), monomer MA, solvents (anisole), $CuBr_2$, and Me_6TREN, molar ratios shown in supporting information, were mixed thoroughly in a sealed Schlenk flask, followed by degassing by bubbling with nitrogen. Then, the $Sn(EH)_2$ was injected into the Schlenk flask to activate the catalyst complex, and the flask was immediately put into an oil bath set at the desired temperature. The conversion was monitored and controlled under 10% by ^{1}H-NMR. The final products were precipitated in cold methanol and then dissolved and stored in THF.

Molar ratios for synthesis of SiO_2-dis-PMA, SiO_2-mid-dis-PMA SiO_2-PMA, and PMA via ARGET ATRP. For SiO_2-*dis*-PMA samples (SiO_2-*dis*-PMA_{400}, SiO_2-*dis*-PMA_{700}, SiO_2-*dis*-PMA_{900}), Initiator (SiO_2-Br, 0.06g), monomer: MA (4.75 g, 5.0 mL), solvents (anisole 9 mL), $CuBr_2$ (2.5×10^{-6} g in 0.05 mL DMF), Me_6TREN (1×10^{-4} mL), and $Sn(EH)_2$ (0.01 g, 0.01mL). For SiO_2-*mid-dis*-PMA_{400}, Initiator (SiO_2-Br, 0.2g), monomer: MA (7.60 g, 8.0 mL), solvents (anisole 4 mL), $CuBr_2$ (2×10^{-4} g in 0.04 mL DMF), Me_6TREN (1×10^{-4} mL), and $Sn(EH)_2$

(0.01 g, 0.01mL). For SiO_2-PMA_{400}, Initiator (SiO2-Br, 0.1g), monomer: MA (4.75 g, 5.0 mL), solvents (anisole 10 mL), $CuBr_2$ (0.005 g in 1 mL DMF), Me_6TREN (0.01 mL), and $Sn(EH)_2$ (0.02 g, 0.02mL). For SiO_2-PMA_{700}, Initiator (SiO_2-Br, 0.08g), monomer: MA (7.60 g, 8.0 mL), solvents (anisole 10 mL), $CuBr_2$ (0.008 g in 1.6 mL DMF), Me_6TREN (0.016 mL), and $Sn(EH)_2$ (0.02 g, 0.02mL). For SiO_2-PMA_{900}, Initiator (SiO_2-Br, 0.05g), monomer: MA (7.60 g, 8.0 mL), solvents (anisole 10 mL), $CuBr_2$ (0.008 g in 1.6 mL DMF), Me_6TREN (0.016 mL), and $Sn(EH)_2$ (0.02 g, 0.02mL). For PMA_{600}, Initiator (EBiB, 0.004 mmol), monomer: MA (7.60 g, 8.0 mL), solvents (anisole 10 mL), $CuBr_2$ (0.008 g in 1.6 mL DMF), Me_6TREN (0.016 mL), and $Sn(EH)_2$ (0.02 g, 0.02mL).

Characterization.

Nuclear Magnetic Resonance Spectroscopy (NMR). Conversion of polymerization was monitored by ^1H NMR on a Bruker Advance 500 MHz NMR instrument in $CDCl_3$ at room temperature.

Size Exclusion Chromatography (SEC). Number-average molecular weights (Mn) and molecular weight distributions (MWD) of samples were determined by size exclusion chromatography (SEC). The SEC was conducted with an Agilent 1260 Iso pump and Waters 410 differential refractometer using PSS columns (Styragel 10^5, 10^3, 10^2 Å) with THF as an eluent at 35 ºC and at a flow rate of 1 mL min^{-1}. Linear PMMA standards were used for calibration. Diphenylethylene and toluene were used as internal standards for the system.

Thermogravimetric Analysis (TGA). TGA with TA Instruments 2950 was used to measure the fraction of SiO_2 in the hybrids. The data were analyzed with TA Universal Analysis. The heating procedure involved four steps: (1) jump to 120 ºC; (2) hold at 120 ºC for 10 min; (3) ramp up at a rate of 20 ºC/min to 800 ºC; (4) hold for 5 min.

Grafting density was calculated using Equation S1.

$$\sigma_{\mathrm{TGA}} = \frac{(1-f_{\mathrm{SiO2}})N_{\mathrm{Av}}\,\rho_{\mathrm{SiO2}}\,d}{6\,f_{\mathrm{SiO2}}\,M_n} \qquad \text{(Equation S1)}$$

where f_{SiO2} is the SiO_2 fraction measured by TGA, N_{Av} is the Avogadro number, ρ_{SiO2} is the density of SiO_2 nanoparticles (2.2 g/cm3), d is the average diameter of SiO_2 nanoparticles (15.8 nm), M_n is the overall number-average MW of the cleaved polymer brushes.

Differential Scanning Calorimetry (DSC). The glass transition temperature (T_g) of linear copolymers were measured by differential scanning calorimetry (DSC) with TA Instrument QA2000. The same procedure was run three times, each involving the following steps: (1) Equilibrate at 25.00 °C, (2) Isothermal for 1.00 min, (3) Ramp 20.00 °C/min to -90.00 °C, (4) Isothermal for 1.00 min, (5) Ramp 20.00 °C/min to 160.00 °C, (6) Isothermal for 1.00 min, (7) Ramp 20.00 °C/min to -90.00 °C, (8) Isothermal for 1.00 min, (9) Ramp 20.00 °C/min to 160.00 °C, (10) Isothermal for 1.00 min, (11) Ramp 20.00 °C/min to -90.00 °C, (12) Isothermal for 1.00 min, (13) Ramp 20.00 °C/min to 160.00 °C, (14) Isothermal for 1.00 min, (15) Jump to 25.00 °C. The DSC data were analyzed with a TA Universal Analysis instrument, and Tg was directly acquired.

Dynamic Mechanical Analysis (DMA). Tensile test: the linear copolymer bulk films are tested in the tensile mode by using DMA (TA RSA-G2). The film thickness was between 100-200 μm. The samples were stretched at a constant tensile rate of 0.05 mm/mm/s at room temperature. Damping property measurement: The damping property was measured through dynamic mechanical analysis (DMA, TA RSA-G2) in a frequency range of 0.1-100 Hz at room temperature, with application of 0.1% strain. All the samples were tested at least three times for consistency. Creep test: Creep experiments were performed on pristine specimens with applied stress of 10 kPa for 90 seconds at room temperature (TA RSA-G2), followed by a recovery time of 180 seconds in which stress was removed.

Shear creep. Shear creep experiments were performed on pristine specimens with applied stress of 100 Pa for 90 seconds at room temperature (Anton Paar MCR 302e).

Cut-and-Adhere Testing. Self-healing of bisected bulk films: a bulk film (dimension: 15 mm x 5 mm x 150 mm) was severed rapidly by a sharp razor blade to get clean-cut surfaces. Then, the two parts were gently rejoined, and two cutting surfaces were softly reattached without any buckling to prevent edges overlap within 1 min and allowed to self-heal for a specific time under ambient conditions. After that time, the same film after healing was characterized by a tensile test as described above. Then, it was compared to the pristine samples and calculated the recovery ratio.

Supporting data

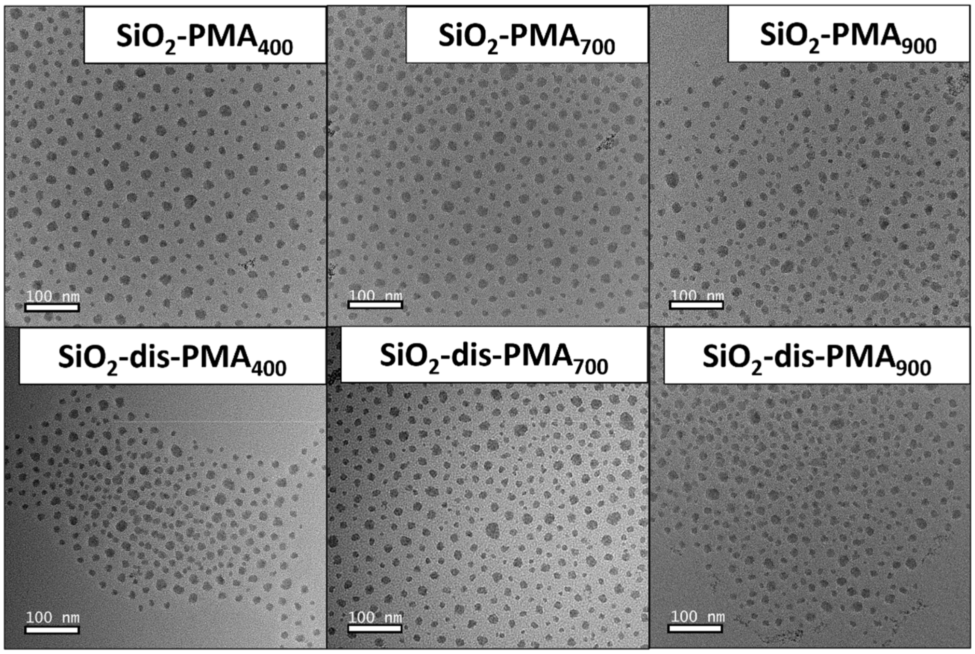

Figure S1. TEM images of SiO_2-g-PMA. (a) SiO_2-dis-PMA-400, (b) SiO_2-dis-PMA-700, (c) SiO_2-dis-PMA-900, (d) SiO_2-PMA-400, (e) SiO_2-PMA-700, (f) SiO_2-PMA-900. Particle diameter determined from analysis of electron micrographs: d ~ 15.54 ± 3.7 nm.

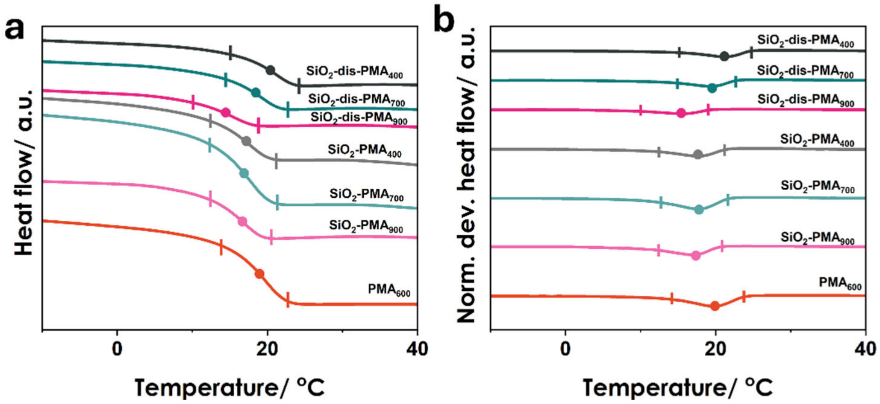

Figure S2. (a) Heat flow curve and (b) normalized derivative heat flow curves. The T_gs are highlighted with solid points, and the T_g onsets and offsets are marked as short solid lines in the figure. All curves were recorded during the 3rd heating/cooling run at a heating rate of 20 ºC min^{-1}.

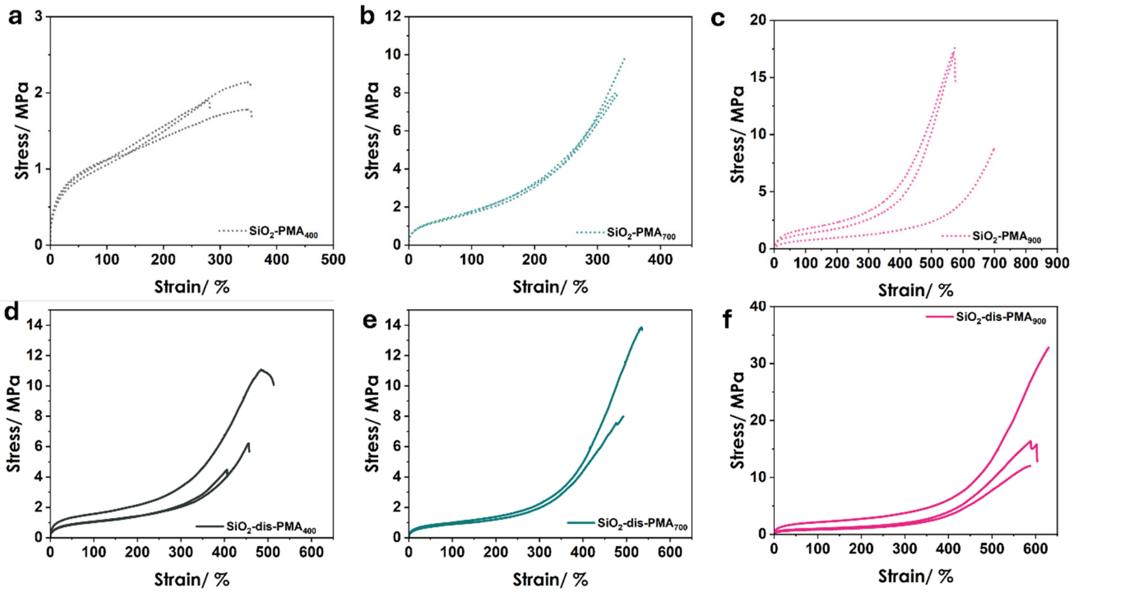

Figure S3. Strain-stress curves: (a) SiO_2-*dis*-PMA_{400}, (b) SiO_2-*dis*-PMA_{700}, (c) SiO_2-*dis*-PMA_{900}, (d) SiO_2-PMA_{400}, (e) SiO_2-PMA_{700}, (f) SiO_2-PMA_{900}. All samples were measured three times with different bulk films as shown in same color lines in the figures.

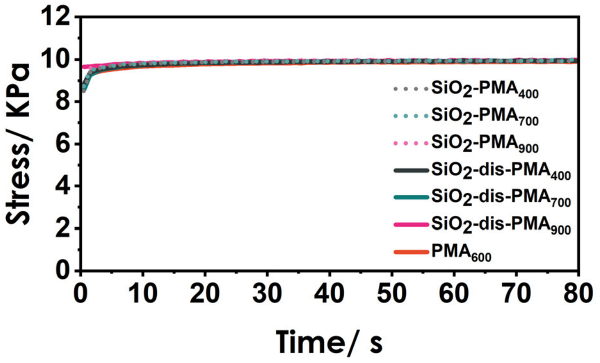

Figure S4. Step-function stress (10 KPa) during creeping testing. Sample dimensions for creep testing: diameter: 25 mm; thickness: 0.2 mm.

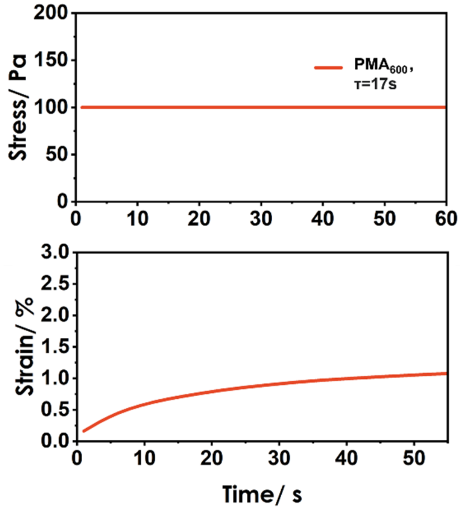

Figure S5. Step-function shear stress (100 Pa, upper plot) and resulting time-dependent shear strain (creep, lower plot) of linear PMA. Sample dimensions for creep testing: diameter: 25 mm; thickness: 0.2 mm.

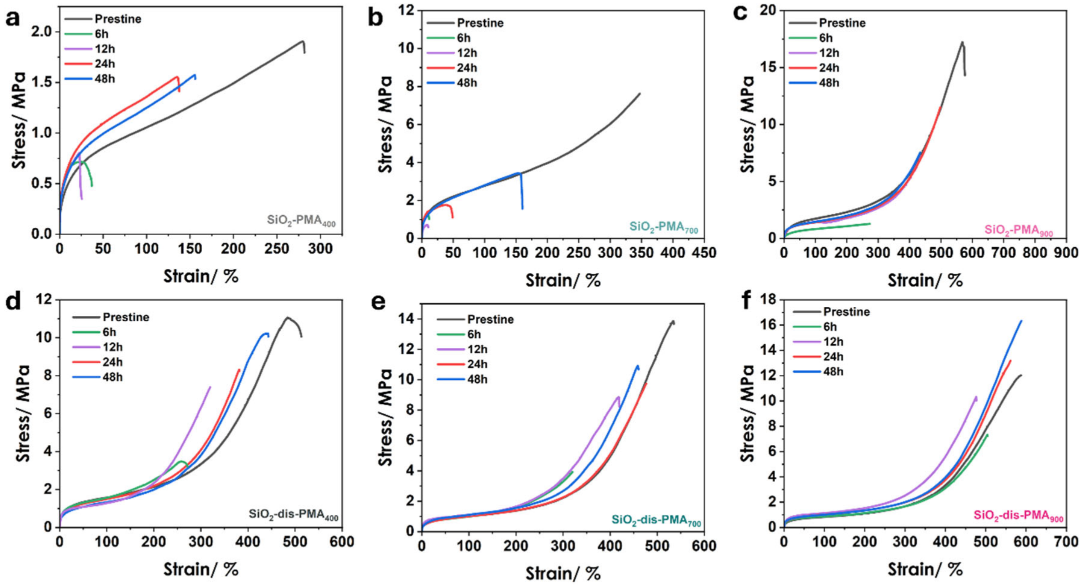

Figure S6. Strain-stress curves for pristine and damaged-and-healed films: (a) SiO_2-dis-PMA-400, (b) SiO_2-dis-PMA-700, (c) SiO_2-dis-PMA-900, (d) SiO_2-PMA-400, (e) SiO_2-PMA-700, (f) SiO_2-PMA-900.

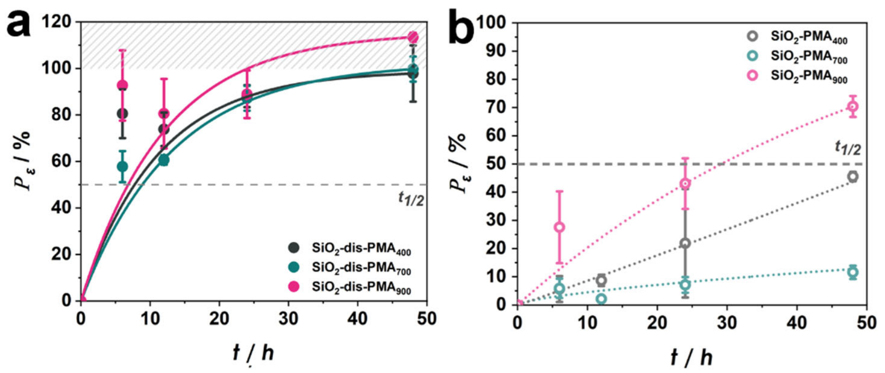

Figure S7. Fractional recovery of strain-to-fracture (Pε) of (a) high dispersity SiO_2-g-PMA samples and (b) low dispersity SiO_2-g-PMA samples after re-joining of films and subsequent annealing at 100 °C. Lines are introduced to guide the eye.

Table S1. Recovery half time.

**Entry**	$t_{1/2E}$ [a]	$t_{1/2U}$ [a]	$t_{1/2\varepsilon}$ [a]
**SiO_2-PMA$_{400}$**	8	>48	>48
**SiO_2-PMA$_{700}$**	3	>48	>48
**SiO_2-PMA$_{900}$**	10	42	30
**SiO_2-*dis*-PMA$_{400}$**	9	9	7
**SiO_2-*dis*-PMA$_{700}$**	7	9	8
**SiO_2-*dis*-PMA$_{900}$**	12	11	7

a: Self-healing half time of fracture toughness calculated by strain–stress curves by the tensile test.

a Characteristics of SiO_2-g-PMA brush particles.

Entry	M_n	M_w/M_n	f_{SiO2}(%)	σ (nm^{-2})	T_g (°C)	T_g Range (°C)
SiO_2-PMA$_{400}$	37,260	1.05	17.17	0.45	17	12~22
SiO_2-*mid-dis*-PMA$_{400}$^{f}	36,840	1.33	16.52	0.47	20	15~22
SiO_2-*dis*-PMA$_{400}$	36,900	2.19	14.18	0.57	21	16~25

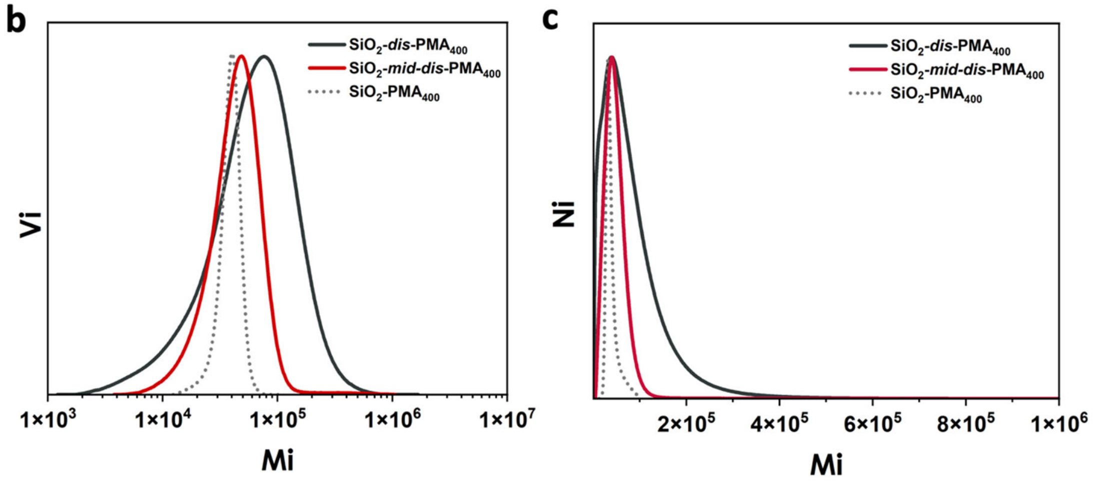

Figure S8. a. Characteristics of SiO_2-g-PMA brush particles with similar Mn and differed dispersity; b. normalized experimental SEC elution curve measured using refractive index detector in THF after etching of SiO2-PMAX using HF; c. normalized number-weighted distribution of molecular weight.

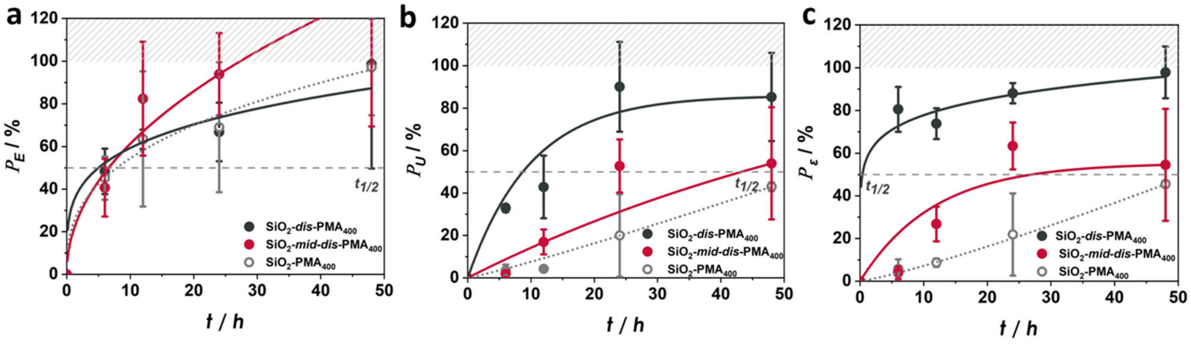

Figure S9. Property recovery of SiO_2-g-PMA$_X$ samples after re-joining films and subsequent annealing at 100 °C. Fractional recovery of (a) Young's modulus (P_E), (b) toughness (P_U) of low- and (c) strain-to-fracture ($P_ε$) of samples with different dispersity.

REFERENCES

(1) Matyjaszewski, K.; Miller, P. J.; Shukla, N.; Immaraporn, B.; Gelman, A.; Luokala, B. B.; Siclovan, T. M.; Kickelbick, G.; Vallant, T.; Hoffmann, H.; et al. Polymers at Interfaces: Using Atom Transfer Radical Polymerization in the Controlled Growth of Homopolymers and Block Copolymers from Silicon Surfaces in the Absence of Untethered Sacrificial Initiator. *Macromolecules* **1999**, *32* (26), 8716–8724. DOI: 10.1021/ma991146.
(2) Jeffrey Pyun; Shijun Jia; Tomasz Kowalewski; Gary D. Patterson; Matyjaszewski, K. Synthesis and Characterization of Organic/Inorganic Hybrid Nanoparticles: Kinetics of Surface-Initiated Atom Transfer Radical Polymerization and Morphology of Hybrid Nanoparticle Ultrathin Films. *Macromolecules* **2003**, *36* (14), 5094–5104. DOI: 10.1021/ma034188.
